# Pharmacokinetics and Bioavailability of a Therapeutic Enzyme (Idursulfase) in Cynomolgus Monkeys after Intrathecal and Intravenous Administration

**DOI:** 10.1371/journal.pone.0122453

**Published:** 2015-04-02

**Authors:** Hongsheng Xie, Jou-Ku Chung, Mary Ann Mascelli, Thomas G. McCauley

**Affiliations:** Shire, Lexington, Massachusetts, United States of America; Hungarian Academy of Sciences, HUNGARY

## Abstract

Intravenous enzyme replacement therapy with iduronate-2-sulfatase is an approved treatment for Hunter syndrome, however, conventional intravenous delivery cannot treat the neurologic manifestations of the disease due to its limited central nervous system penetration. Intrathecal administration of iduronate-2-sulfatase for delivery to the central nervous system is currently under investigation. The objective of this study was to evaluate the pharmacokinetics of idursulfase in the central nervous system of cynomolgus monkeys (*Macaca fasicularis*) after intravenous and intrathecal administration. Twenty-seven monkeys, treatment-naïve to enzyme replacement therapy, were placed into 4 groups according to body weight: Group 1 was administered 0.5 mg/kg idursulfase intravenously, Groups 2–4 were administered an intrathecal formulation (1-, 10-, and 30-mg doses). Blood samples and cerebrospinal fluid (sampled at the cisterna magna or lumbar level) were collected at the same time points for 72 hours post dosing. Following intravenous administration, a high maximum serum concentration and rapid distribution of iduronate-2-sulfatase out of the central compartment were observed (elimination half-life: 4.3 hours). Iduronate-2-sulfatase exposure in the cerebrospinal fluid was limited, suggesting intravenous administration provided minimal penetration of the blood–brain barrier. Following intrathecal administration, a high maximum observed concentration was immediately noted and elimination half-life ranged between 7.8–10 hours and 5.9–6.7 hours (cisterna magna and lumbar sampling, respectively). Cerebrospinal fluid pharmacokinetic profiles at different doses of iduronate-2-sulfatase were similar and the dose/exposure relationship was proportional. After intrathecal administration, movement of iduronate-2-sulfatase from cerebrospinal fluid to serum was observed (systemic bioavailability was 40–83%). The clear penetration of iduronate-2-sulfatase into the cerebrospinal fluid and the dose response suggest that intrathecal delivery of iduronate-2-sulfatase may be suitable for treating the central nervous system manifestations associated with Hunter syndrome.

## INTRODUCTION

Hunter syndrome is a rare (prevalence 1 in 162,000 male live births [1]), X-linked disease caused by a deficiency or absence of the enzyme iduronate-2-sulfatase (I2S), a lysosomal storage enzyme required for the degradation of glycosaminoglycans [2]. When the enzyme is deficient, the glycosaminoglycans heparan sulfate and dermatan sulfate accumulate in the lysosomes, causing the cells to enlarge, leading to organ failure, tissue dysfunction, and reduced life expectancy. In the severe form of the disease there is extensive neurologic involvement and death usually occurs between 10 and 15 years of age [3].

Intravenous enzyme replacement therapy with recombinant human I2S (idursulfase (Elaprase) Shire, Lexington, MA) is an approved treatment for Hunter syndrome [4] and is currently available in over 50 countries. Conventional enzyme replacement therapy with idursulfase administered intravenously is unlikely to enter the brain in sufficient amounts to treat the often very serious central nervous system (CNS) manifestations of Hunter syndrome [5]. This is a major challenge for the treatment of patients with Hunter syndrome who have CNS manifestations and is a problem common to many life-threatening CNS diseases, where an effective method of administering drug therapy to the brain is unavailable.

The cynomolgus monkey was chosen as the test system for this study because of its established usefulness and acceptance as a model for toxicological and pharmacological studies in a large animal species.

Studies by Felice et al have demonstrated that monthly administration, using a formulation of idursulfase appropriate for intrathecal-lumbar (IT-L) administration (idursulfase-IT) in addition to weekly idursulfase-intravenous infusions was generally well tolerated in cynomolgus monkeys for 6 months [6]. They also observed that I2S was distributed throughout the brain and spinal cord, similar to what has been seen in other IT enzyme replacement therapy studies in mucopolysaccharidosis I dog and mucopolysaccharidosis IIIA mouse models [7]. A later study [8] demonstrated that intracerebroventricular and IT-L administration of idursulfase-IT to dogs and cynomolgus monkeys resulted in the enzyme being detected widely throughout the brain. They found that the distribution of the enzyme was very similar, irrespective of whether the IT-L or intracerebroventricular administration method was used. IT-L administration has a number of advantages over intracerebroventricular administration: firstly, it is less invasive; secondly, it is a routine clinical procedure; and finally, spinal administration may help alleviate the glycosaminoglycan deposition in the spinal cord itself. In an *I2S* gene knockout mouse model of Hunter syndrome, Calias et al also found that IT-L–delivered idursulfase treatment produced a decrease in cellular vacuolation in the surface cerebral cortex, caudate nucleus, thalamus, cerebellum, and white matter compared to untreated mice, indicating that I2S was active within the neural tissue [8].

The objectives of this study were to investigate the pharmacokinetic behaviours of idursulfase after intravenous and IT-L administration, the dynamics of idursulfase transfer in the cerebrospinal fluid (CSF), and the dynamics of idursulfase movement from the CSF to the systemic circulation in cynomolgus monkeys.

## MATERIALS AND METHODS

### Ethics Statement

Care of the animals was conducted in accordance with the guidelines *Guide for the Care and Use of Laboratory Animals*, *United States Department of Health and Human Services*, *No 86–23*, and the *Animal Welfare Act (9 CFR Part 3); USDA No*. *34-R-0025)*. The studies were performed at Northern Biomedical Research, Inc. (Muskegon, Michigan)(NBR), an organization accredited by the Association for Assessment and Accreditation of Laboratory Animal Care. The studies were approved by the Institutional Animal Care and Use Committee of NBR (cynomolgus monkey study protocol number 047–028).

### Experimental Animals

#### Housing and Husbandry

Cynomolgus monkeys (*Macaca fasicularis*)(Covance Research Products, Emeryville, CA) were singly housed in a colony room under a 12-h light-dark cycle, 50% (± 30%) humidity, and 22°C (± 2°C). Appropriate food, water, treats, and vitamin supplements were provided, and animals were given access to environmental enrichment such as approved toys, swings, perches, mirrors, television, or music to promote psychological well-being. Every effort was made to minimize pain, discomfort, and suffering through the use of appropriate methods and agents for analgesia, anesthesia, and euthanasia. All animals were under the care and supervision of a veterinarian.

The animals were individually housed in stainless steel cages in two rooms at NBR. Temperature and humidity were recorded daily. The temperature and relative humidity values of the animal rooms were 20°-23°C (68°-73°F) and 41%-78% for one room, and 19°-24°C (66–75°F) and 27%-69% for the other. Each room was equipped with an automatic timer and the animals received 12 hours of light and darkness each day. The light-dark cycle was interrupted for additional observations or procedures; these interruptions were recorded in the raw data. Room airflow was set to provide at least 10 air changes per hour. Excrement pans were cleaned and fresh wood chips were added daily. The animal cages were washed and sanitized every two weeks. The animals received environmental enrichment per the current NBR Program of Animal Care.

Twenty-five biscuits of PMI Certified Primate Diet 5048 (PMI Nutrition, Arden Hills, Minnesota) were placed in the animals’ feeders each day. The manufacturer analyzed the feed and no contaminants were shown to exist in the food at levels that would be expected to interfere with the integrity of this study. A copy of the feed analysis for each feed lot used on study is kept in the study records. The animals’ diet was supplemented with vitamin C (manufacturer and lot numbers were recorded in the raw data) on a weekly basis.

Food consumption data was collected daily beginning prior to surgery and continued throughout the dosing period. Food was withheld at least 12 hours prior to general anesthesia (e.g., surgery). With the approval of the study director, food was not withheld 12 hours prior to repair surgeries.

The water utilized on this study (Muskegon municipal water) was provided via a filtered automatic water system *ad libitum*. The water was analyzed annually for heavy metals, trihalomethanes, PCBs and pesticides, and analyzed quarterly for microbiological contaminants. Copies of the water analyses conducted during the course of this study are maintained in the study notebook. No contaminants were shown in the water at concentrations expected to interfere with the purpose of this study.

No animals were sacrificed during this study. Animals were observed at least twice daily for morbidity and mortality beginning on the first day of dosing. No idursulfase-related morbidity or mortality was observed during the study. Clinical signs were recorded at least twice daily post surgery throughout the study. The animals were observed for signs of clinical effects, illness, and/or death. Clinical signs unrelated to idursulfase included wounds (i.e. wound on back of neck from collar, wound on back of head), incision/delivery system observations (i.e. seroma over cisterna magna port, reddened area over lumbar port), paresis, motor deficit, loose stool and emesis.

Incision/delivery system observations were noted for various animals. The majority of the observations were noted in the week prior to the dosing period. Wounds were noted for a few of the animals on one to five days throughout the study.

Clinical observations noted included paresis (Days -7 to -4), motor deficit (Days -3 to 3, 6, and 7), incision/delivery system observations (Day -2), few feces (Day -5), and wounds (Days 4 to 6) occurring in one animal each.

### Study Design

Twenty-seven treatment-naïve cynomolgus monkeys (13 males and 14 females) were assigned to four groups based on their body weights (Table 1). Randomization was based on body weight as brain size is correlated with body weight. The doses selected for this study were based on the human equivalent doses, the scaling factor based on brain weight. As there is approximately a tenfold difference in brain weight between monkeys and humans, doses of 1, 10 and 30 mg idursulfase-IT in the monkey were considered equivalent to human doses of approximately 10, 100 and 300 mg. Both investigators and sponsor were blinded. The number of animals chosen for the study was based on the minimum required for pharmacokinetic evaluation. Animals in Group 1 were administered idursulfase via intravenous administration, and Groups 2–4 were administered idursulfase-IT via IT-L administration. The doses tested in this study bracket the range of anticipated potential clinical doses for human administration. The IT-L route of administration was selected because this is the anticipated route for human administration. The intravenous route of administration was selected to allow calculation of absolute bioavailability after IT-L administration. The number of independent replications of each experiment was 3 or 4 per gender (given the high inter-animal variability).

**Table 1:** Cynomolgus dosing and administration route groups.

	**Number and body weight of animals**							
**Group**	**Male, n**	**Mean weight (±SD), kg**	**Female, n**	**Mean weight (±SD), kg**	**Nominal dose concentration (mg/ml)**	**Dose**	**I2S**	**Administration Route**
1	3	2.70 (±0.07)	4	2.71 (±0.06)	2	0.5 mg/kg	idursulfase-IV	IV
2	3	3.10 (±0.85)	3	2.63 (±0.19)	1	1 mg	idursulfase-IT	IT-L
3	3	2.73 (±0.22)	4	2.82 (±0.25)	10	10 mg	idursulfase-IT	IT-L
4	4	2.89 (±0.10)	3	2.66 (±0.20)	30	30 mg	idursulfase-IT	IT-L

IT-L, intrathecal lumbar; IV, intravenous, SD, standard deviation.

### Experimental Procedures

#### Surgery and Surgical Recovery

At catheter implantation, the animals were pretreated with a subcutaneous injection of atropine sulfate at a dose of 0.04 mg/kg. Approximately 15 minutes later, an intramuscular (IM) dose of 8 mg/kg of ketamine HCl was provided to induce sedation. The animals were masked to a surgical plane of anesthesia, intubated, and maintained on approximately 1 L/min of oxygen and 2% isoflurane. The anesthetic gases and mixtures varied as required by each individual animal; these variations were recorded in the raw data. Prednisolone sodium succinate IV, 30 mg/kg, and flunixin meglumine IM, 2 mg/kg, were administered prior to surgery.

For IT-L catheter implantation, an incision was made over the dorsal process of the lumbar spine at L5 or L6. The muscle was dissected and a hemilaminectomy was made for the insertion of a tapered polyurethane catheter (0.9 mm OD and 0.5 mm ID tapered/sheathed open end catheter with side holes, P/N 69–2320). The catheter was advanced to the area of the thoraco-lumbar junction. The proximal end of the IT-L catheter was attached to a subcutaneous access port (P.A.S. PORT Elite plastic/titanium portal with ULTRA-LOCK connector, P/N 69–2316). Proper catheter placement was confirmed with the aid of a myelogram with Isovue 300 (0.8 ml, Bracco Diagnostics, Inc, Princeton, New Jersey). The tissue layers were closed with sutures and tissue adhesive was applied.

For sampling the CSF at the cisterna magna (CM) level, a dorsal sagittal incision was made over the caudal calvarium and rostral cervical area. The musculature was dissected and a hemilaminectomy of C2 was performed. A very small durotomy was made to prevent CSF leakage and a polyurethane catheter (0.9 mm OD and 0.5 mm ID tapered/sheathed open end catheter with side holes, P/N 69–2320) introduced approximately 2 cm anterograde into the subarachnoid space. The proximal end of the CM catheter was attached to a subcutaneous access port (P.A.S. PORT Elite plastic/titanium portal with ULTRA-LOCK connector, P/N 69–2316). The tissue layers were closed with sutures and tissue adhesive was applied.

Upon recovery from anesthesia, the animals were provided butorphanol tartrate IM, 0.05 mg/kg, for analgesia and placed on post-surgical antibiotic ceftiofur sodium IM, 5.0 mg/kg, b.i.d. (one injection prior to surgery followed by three injections). Dosing started at least seven days after surgery.

#### Idursulfase

Recombinant human I2S was expressed and purified from a human-derived cell line [4] and was provided in IT formulation (idursulfase-IT) [6]. This was diluted with 154 mm NaCl, 0.005% polysorbate 20, pH 6.0 to bring to a concentration of 30 mg/ml and stored at -60°C. The formulation was warmed to room temperature on the bench top before using. For intravenously administered idursulfase, the commercially available idursulfase formulation was used.

#### Administration of idursulfase-IT

Idursulfase-IT was administered intrathecally through a catheter implanted at the lumbar spine level. An IT-L catheter (P/N 69–2320) was implanted in each animal in Groups 2, 3, and 4 through an incision over the dorsal process of the lumbar spine L_5_ or L_6_.

The IT-L dosing was performed by hand and the rate of solubilization of the manual bolus was approximately 0.5 mL over one minute. The dose volume was 1.0 mL per animal, followed by 0.5 mL of phosphate buffered saline to flush the dose from the catheter system. The total duration of administration was approximately 2–3 minutes. Each animal received 1 mg (Group 2), 10 mg (Group 3), or 30 mg of idursulfase-IT (Group 4). The low-dose group were treated and assessed first, followed by the higher-dose groups.

#### CSF and blood sampling

CSF samples (~0.1 ml) were collected after intravenous and IT-L dosing from the CM catheter (n = 4, 2 males/2 females from each of the 4 groups) at pre-dose (time 0), 5 min, 15 min, 30 min, 1, 2, 4, 8, 24, 48, and 72 h post dosing. The animals were not sedated for any sampling procedure: all IT-L and CM samples were taken while the animals were awake. In order to study the movement of the dosed protein in the CSF, CSF samples of the same volume were also collected from the IT-L catheter that had been used for IT-L dosing of idursulfase-IT (n = 3 in Groups 1, 3, and 4; and n = 2 in Group 2) at 4, 8, 24, 48, and 72 h post dose.

Blood (~1 ml) samples were collected at the same time points as described for CSF sampling. Serum samples were prepared by clotting at room temperature, and then centrifuged for 15 min.

#### CSF and Serum Assays

The concentrations of I2S from the serum and CSF samples were analyzed by an I2S-specific ELISA technique at Covance Laboratories, Inc. (Immunochemistry Services, 3635 Concorde Parkway, Ste 100, Chantilly, VA 20151–1130). The capture antibody was a polyclonal goat anti-idursulfase IgG

(with some cross-reactivity to monkey I2S), and the detection antibody was a horseradish peroxidase-conjugate of the same goat anti-idursulfase IgG. Samples exceeding the high end of the calibration curve were further diluted and retested. The concentrations were expressed as ng/ml, and the validated lower limit of quantitation at a 1:50 dilution was 62.5 ng/ml.

#### Pharmacokinetic Assessments

Serum and CSF concentration-time data from individual animals were analyzed using a non-compartmental analysis method as implemented in the WinNonlin 5.2 program (Pharsight Corp., Mountain View, CA). The following pharmacokinetic parameters were observed or calculated: maximum observed serum concentration (*C*_max_); time of *C*_max_ (*T*_max_); area under the serum concentration-time curve from time 0 to the last sampling time at which serum concentrations were measurable (*AUC*_0-t_) as calculated by the linear up/logarithmic down trapezoidal summation method; area under the serum concentration-time curve extrapolated to infinity (*AUC*_0-inf_) calculated by the linear up/logarithmic down trapezoidal summation method; apparent terminal rate constant (λZ) derived from the slope of the log-linear regression of the log-linear terminal portion of the plasma concentration-time curve; mean residual time derived from time 0 to infinity (*MRT*_inf_); terminal half-life (*t*_½_) calculated as 0.693/λZ; apparent volume of distribution (V or V/F) calculated as dose/(λZ * AUC_inf_); total clearance (CL) calculated as dose/*AUC*_inf_; bioavailability (F) of IT (or IV) idursulfase relative to the IV (or IT) reference estimated as the ratio of AUC(AUC_inf_IT (or IV)_/AUC_inf_IV (or IT_)) * 100%;

## RESULTS

### I2S in the serum

**Fig 1:**
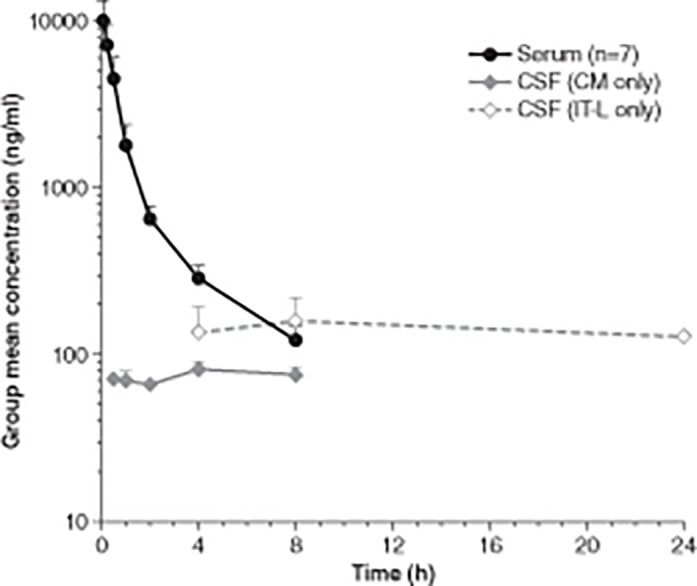
Mean serum and CSF I2S concentration-time profiles with 0.5 mg/kg idursulfase intravenous dose. Error bars represent standard deviation.CM, cisterna magna; CSF, cerebrospinal fluid; I2S, iduronate-2-sulfatase; IT-L, intrathecal lumbar.

The mean serum and CSF I2S concentration-time profiles of an intravenous injection of 0.5 mg/kg idursulfase are shown in Fig 1. Serum I2S concentrations declined rapidly from a peak of 10,029 ± 3,127 ng/ml and the last time point with a measurable concentration (122 ± 26 ng/ml) was 8 h after dosing. The serum I2S pharmacokinetic parameters are listed in Table 2. Serum total clearance for intravenously administered idursulfase was 57.5 ± 22.4 ml/h/kg with a elimination half-life (*t*_1/2_) 4.3 ± 4.4 h, and the mean residual time derived from time 0 to infinity (*MRT*_inf_) was 4.8 ± 7.4 h. The volume of distribution at steady state (*V*_ss_) was 198 ± 191 ml/kg.

**Table 2:** Comparisons of serum I2S pharmacokinetic parameters after 4 different idursulfase treatments.

	**IV dose**		**IT-L dose**					
	**0.5 mg/kg**		**1 mg**		**10 mg**		**30 mg**	
	**(n = 7)**		**(n = 4)**		**(n = 6)**		**(n = 7)**	
**Parameter**	**Mean**	**(±SD)**	**Mean**	**(±SD)**	**Mean**	**(±SD)**	**Mean**	**(±SD)**
λ_Z_ (1/h)	0.238	(±0.096)	0.220	(±0.107)	0.094	(±0.055)	0.086	(±0.027)
*t*_½_ (h)	4.3	(±4.4)	4.2	(±3.0)	10.2	(±6.5)	9.0	(±3.4)
*T*_max_ (h)	0.1	(±0)	2.8^b^	(±1.3)	3.4^c^	(±2.2)	4.0	(±0)
*C*_max_ (ng/ml)	10,029	(±3127)	296^b^	(±155)	4740^c^	(±1574)	19,971	(±4508)
*AUC*_0-t_ (h*ng/ml)	9404	(±4340)	1745^b^	(±1347)	36,436^c^	(±8152)	176,818	(±47,077)
*AUC*_inf_ (h*ng/ml)	10,074	(±4762)	2777	(±1642)	37,618	(±9145)	178,042	(±47,436)
*V*_z_ (ml)^a^	290	(±164)	2410	(±1111)	4016	(±2412)	2207	(±606)
*V*_ss_ (ml)	198	(±191)	na		na		na	
CL (ml/h)^d^	57.5	(±22.4)	563.4	(±513.5)	280.9	(±75.5)	178.5	(±44.1)
*MRT*_inf_ (h)	4.8	(±7.4)	6.7	(±4.3)	9.7	(±4.2)	8.5	(±1.2)

*AUC*_inf_, area under the concentration-time curve extrapolated to infinity; *AUC*_0-t_, area under the concentration-time curve from time 0 to the last sampling time; CL, total clearance; *C*_max_, maximum observed concentration; I2S, iduronate-2-sulfatase; IT-L, intrathecal lumbar; IV, intravenous; *MRT*_inf_, mean residual time derived from time 0 to infinity; na, not available; SD, standard deviation; *t*_½_, terminal half-life; *T*_max_, time of occurrence of *C*_max_; *V*_ss_, volume of distribution at steady state; *V*_z_, volume of distribution during terminal phase; λ_Z_, apparent terminal rate constant.

^a^Units were ml/kg for IV dosing and ml for IT-L

^b^n = 6

^c^n = 7

^d^Units were ml/h/kg for IV dosing and ml/h for IT-L.

**Fig 2:**
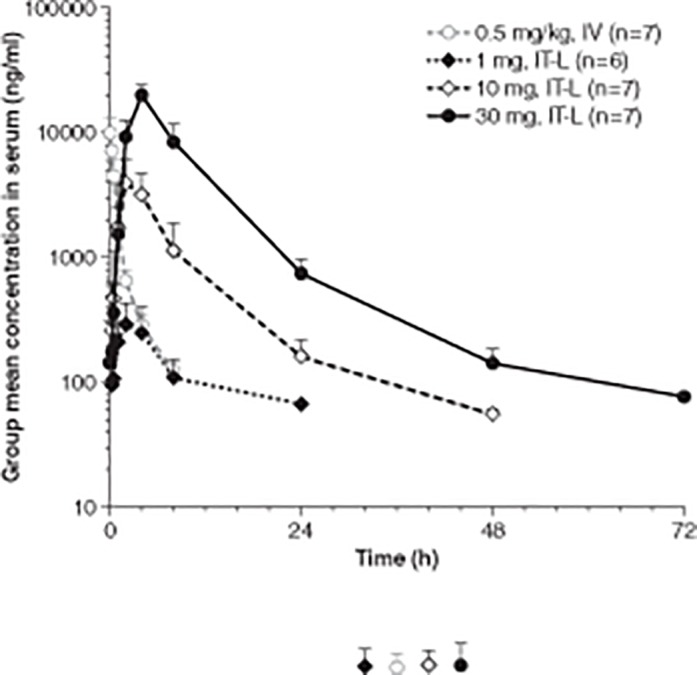
Mean intravenous and IT-L serum I2S concentration-time profiles. Error bars represent standard deviation.I2S, iduronate-2-sulfatase; IT-L, intrathecal lumbar; IV, intravenous.

I2S was detected in the serum at the earliest time point (5 min) after IT-L administration of idursulfase-IT in 9/20 animals. I2S serum concentrations increased rapidly and reached maximum observed concentration (*C*_max_) approximately 3–4 h after IT-L administration (Fig 2; Table 2). The serum C_max_ for the 30-mg dose of idursulfase-IT was 19,971 ± 4,508 ng/ml, approximately twice that of the intravenously administered dose of idursulfase (10,029 ± 3,127 ng/ml). Serum concentrations were detectable (108 ± 44 ng/ml) in 5/6 animals for 8 h post 1 mg IT-L dosing. I2S remained measurable (203 ± 71 ng/ml) for 24 h in all animals and for 48 h in 4/7 animals (81 ± 17 ng/ml) post 10 mg IT-L dosing. Measurable concentrations of I2S (141 ± 44 ng/ml) were detected up to 48 h in all animals and up to 72 h in 3/7 animals (77 ± 7 ng/ml) post 30 mg IT-L dosing.

Following IT-L dosing, serum I2S total clearance values were 563.4 ± 513.5, 280.9 ±75.5, and 178.5 ±44.1 ml/h/kg after 1-, 10-, and 30-mg doses of idursulfase-IT, respectively. The observation that total clearance decreases with the increased idursulfase-IT doses, together with the greater than dose-proportional serum exposure after IT-L administration, suggests a potential saturated mechanism of binding sites in the peripheral tissues (Table 2).

### I2S in the CSF

After intravenous dosing, sampled from the CM port, I2S concentrations in the CSF were measurable from 0.5 to 8 h in 3 out of 4 monkeys. These concentrations remained in a range of 62.6 ng/ml to 87.6 ng/ml, suggesting a minimal translocation of I2S from serum to CSF after intravenous dosing (Fig 1). Total clearance, *t*_1/2_,*V*_ss_, and the area under the concentration-time curve from time 0 to infinity (*AUC*_inf_), could not be precisely determined due to the lack of sufficient numbers of I2S serum concentration-time values needed to derive these pharmacokinetic parameters. The *C*_max_, time of occurrence of *C*_max_ (*T*_max_), and the area under the concentration-time curve from time 0 to last sampling time with measurable concentration (*AUC*_0-t_) were evaluated (n = 3) [Table 3]). After intravenous dosing, the *T*_max_ of I2S in CSF was 4.2 ± 3.8 h, the *C*_max_ was 80 ± 8 ng/ml, and the *AUC*_0-t_ was 390 ± 242 h∙ng/ml.

**Table 3:** CSF pharmacokinetic parameters of idursulfase IV and IT at CM level.

	**IV dose**		**IT-L dose**					
	**0.5 mg/kg**		**1 mg**		**10 mg**		**30 mg**	
	**(n = 1)**		**(n = 4)**		**(n = 4)**		**(n = 4)**	
**Parameter**	**Mean**	**(±SD)**	**Mean**	**(±SD)**	**Mean**	**(±SD)**	**Mean**	**(±SD)**
λ_Z_ (1/h)	0.063	na	0.109	(±0.068)	0.100	(±0.046)	0.080	(±0.032)
*t*_½_ (h)	11.1	na	9.5	(±7.9)	7.8	(±2.9)	10.0	(±4.7)
*T*_max_ (h)	4.2^b^	(±3.8)	0.083	(±0)	0.14	(±0.10)	0.083	(±0)
*C*_max_ (ng/ml)	80^b^	(±8)	261,333	(±16,289)	2,960,000	(±1,227,477)	9,010,000	(±2,561,744)
*AUC*_0-t_ (h*ng/ml)	390^b^	(±242)	368,082	(±81,429)	4,566,101	(±665,883)	16,446,859	(±2,549,015)
*AUC*_inf_ (h*ng/ml)	1130	na	369,511	(±81,349)	4,570,535	(±667,976)	16,484,049	(±2,516,095)
*V*_z_ (ml)^a^	7054	na	35	(±25)	24	(±7)	28	(±17)
*V*_ss_ (ml)	-	-	8.5	(±0.9)	9.1	(±2.7)	9.2	(±1.9)
CL (ml/h)^c^	442	na	2.8	(±0.7)	2.2	(±0.3)	1.9	(±0.3)
*MRT*_inf_ (h)	16	na	3.2	(±0.9)	4.2	(±1.6)	5.0	(±0.5)

*AUC*_inf_, area under the concentration-time curve extrapolated to infinity; *AUC*_0-t_, area under the concentration-time curve from time 0 to the last sampling time; CL, total clearance; CM, cisterna magna; *C*_max,_ maximum observed concentration; CSF, cerebrospinal fluid; I2S, iduronate-2-sulfatase; IT-L, intrathecal lumbar; IV, intravenous; *MRT*_inf,_ mean residual time derived from time 0 to infinity; na, not available; SD, standard deviation; *t*_½_, terminal half-life; *T*_max_, time of occurrence of *C*_max_; *V*_ss_, volume of distribution at steady state; *V*_z_, volume of distribution during terminal phase; λ_Z_, apparent terminal rate constant.

^a^Units were ml/kg for IV and ml for IT-L dosing

^b^n = 3

^c^Units were ml/h/kg for IV dosing and ml/h for IT-L.

After intravenous dosing, sampled from the lumbar port, I2S concentrations in the CSF were measurable from 4 to 24 h in 2 out of 3 monkeys. These concentrations remained in a range of 92.9 ng/ml to 202 ng/ml, suggesting a minimal translocation of I2S from serum to CSF after intravenous dosing (Fig 1). Total clearance, *t*_1/2_,*V*_ss_, and *AUC*_inf_, could not be determined due to the lack of sufficient numbers of I2S serum concentration-time values needed to derive these PK parameters. The *T*_max_ of I2s in CSF was 8 h, the *C*_max_ was 159 ng/ml, and the *AUC*_0-t_ was 2176 h∙ng/ml (n = 2)(Table 4).

**Table 4:** CSF pharmacokinetic parameters of idursulfase IV and IT at lumbar level.

	**IV dose**		**IT-L dose**					
	**0.5 mg/kg**		**1 mg**		**10 mg**		**30 mg**	
	**(n = 2)**		**(n = 2)**		**(n = 3)**		**(n = 3)**	
**Parameter**	**Mean**	**(±SD)**	**Mean**	**(±SD)**	**Mean**	**(±SD)**	**Mean**	**(±SD)**
λ_Z_ (1/h)	na	(na)	0.118	(±0.007)	0.109	(±0.015)	0.104	(±0.008)
*t*_1/2_ (h)	na	(na)	5.9	(±0.3)	6.5	(±0.9)	6.7	(±0.5)
*T*_max_ (h)	8.0	(±0)	4.0	(±0)	4.0	(±0)	2.8	(±2.2)
*C*_max_ (ng/ml)	159	(±61)	139,000	(±96,167)	1,330,000	(±310,483)	4,790,000	(±2,418,202)
*AUC*_0-t_ (h*ng/ml)	2176	(±2223)	2,735,309	(±1,689,056)	20,723,722	(±5,506,951)	44,117,739	(±10,853,915)
*AUC*_inf_ (h*ng/ml)	na	(na)	2,736,552	(±1,689,808)	20,728,812	(±5,504,098)	44,124,585	(±10,853,793)
*V*_z_ (ml)^a^	na	(na)	4	(±3)	5	(±2)	7	(±2)
*V*_ss_ (ml)	na	(na)	0.7	(±0.3)	2.6	(±1.7)	2.9	(±0.9)
CL (ml/h)^b^	na	(na)	0.5	(±0.3)	0.5	(±0.1)	0.7	(±0.2)
*MRT*_inf_ (h)	na	(na)	1.6	(±0.4)	4.8	(±2.2)	4.1	(±1.1)

*AUC*_inf_, area under the concentration-time curve extrapolated to infinity; *AUC*_0-t_, area under the concentration-time curve from time 0 to the last sampling time; CL, total clearance; *C*_max,_ maximum observed concentration; CSF, cerebrospinal fluid; I2S, iduronate-2-sulfatase; IT-L, intrathecal lumbar; IV, intravenous; *MRT*_inf,_ mean residual time derived from time 0 to infinity; na, not available; *t*_½_, terminal half-life; *T*_max_, time of occurrence of *C*_max_; *V*_ss_, volume of distribution at steady state; *V*_z_, volume of distribution during terminal phase; λ_Z_, apparent terminal rate constant.

^a^Units were ml/kg for IV and ml for IT-L dosing

^b^Units were ml/h/kg for IV dosing and ml/h for IT-L.

**Fig 3:**
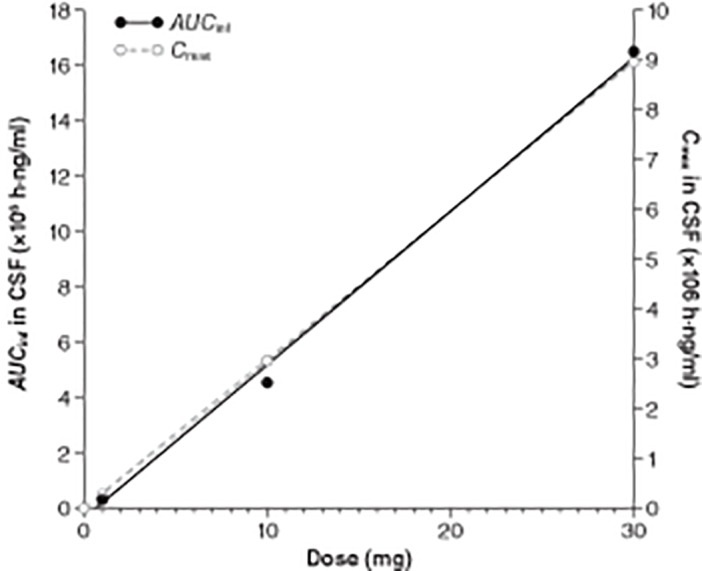
Proportional relationship between idursulfase-IT dose and CSF I2S *AUC*_inf_ and *C*_max_. *AUC*_inf_, area under the serum concentration-time curve extrapolated to infinity; *C*_max,_ maximum observed concentration; CSF, cerebrospinal fluid; I2S, iduronate-2-sulfatase.

The dynamics of I2S distribution in the CSF was evaluated by comparing the pharmacokinetics of CSF samples collected from the CM level to the lumbar level to evaluate the potential slow disposition of I2S in the CSF. Idursulfase-IT was administered at the lumbar level, but CSF sampling was done at both the lumbar and the CM levels, at simultaneous time points. Peak concentrations (*C*_max_) of I2S were 261,333 ± 16,289, 2,960,000 ± 1,227,477, and 9,010,000 ± 2,561,744 ng/ml for the 1-, 10-, and 30- mg IT-L treated groups, respectively, in the CSF samples taken at the CM level 5 min after IT-L administration (Table 3). These peak concentrations rapidly decreased to 3804 ± 3174 (69-fold decrease), 62,098 ± 47,479 (48-fold), and 316,750 ± 102,679 (28-fold) ng/ml at 8 h following dosing of 1, 10, and 30 mg, respectively. Thereafter, CSF concentrations decreased slowly, following approximately first-order kinetics. The CSF concentrations were still measurable for 72 h after IT-L dosing in all 3 groups (75 [n = 1], 451 ±173 [n = 2], and 2135 ± 1375 [n = 4] ng/ml in 1-, 10-, and 30-mg treated groups, respectively). Following IT-L dosing, *C*_max_ values in the CSF increased dose-proportionally (10-, 3-, and 30-fold increase in IT dose with 11-, 3-, and 35-fold increase in *C*_max_). *AUC*_inf_ values increased 12-, 4-, and 45-fold with the increasing dose (1, 10, and 30 mg, respectively). These *AUC*_inf_ values indicate a slightly larger than dose-proportional increase in CSF exposure (Tables 3 and 4; Fig 3).

Following IT-L dosing, mean total clearance of I2S from the CSF was 2.3 ml/h (2.8 ± 0.7 ml/h, 2.2 ± 0.3 ml/h, and 1.9 ± 0.3 ml/h for 1-, 10-, and 30-mg treated groups, respectively), which is similar to physiological clearance volume of the CSF in monkeys (2.4 ml/h). The mean *t*_1/2_ was 9.1 h (9.5 ± 7.9 h, 7.8 ± 2.9 h, and 10.0 ± 4.7 h for 1-, 10-, and 30-mg treated groups, respectively), and mean *MRT*_inf_ was 4.1 h (3.2 ± 0.9, 4.2 ± 1.6, and 5.0 ± 0.5 h, respectively). The mean *V*_ss_ was 8.9 ml (8.5 ± 0.9, 9.1 ± 2.7, and 9.2 ± 1.9 ml for these corresponding groups).

**Fig 4:**
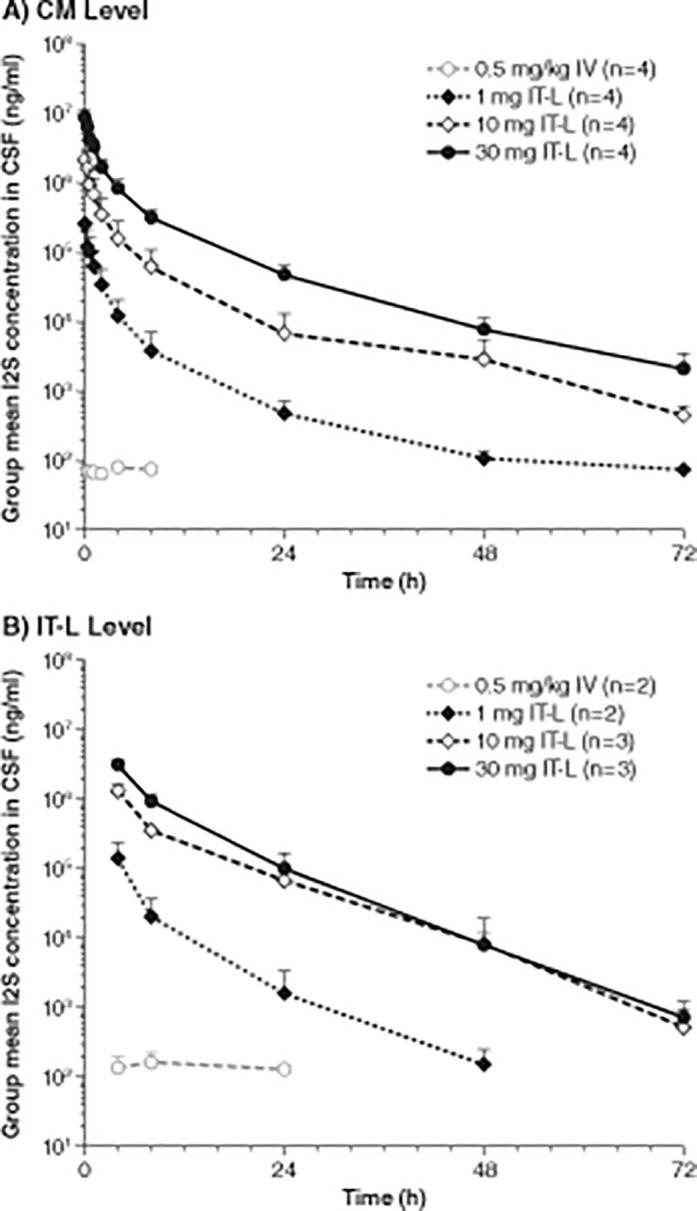
Mean CSF I2S concentration-time profiles. After intravenous and IT-L dosing, mean CSF I2S concentration-time profiles were determinedwith CSF sampled at the CM level (A) and at the IT-L level (B). Error bars represent standard deviation. CSF, cerebrospinal fluid; I2S, iduronate-2-sulfatase; IT-L, intrathecal lumbar; IV, intravenous.

I2S concentrations in the CSF samples taken at the lumbar level from 4 to 72 h were significantly higher than those taken at the CM level at the same time points after IT-L dosing (Fig 4A and 4B). The ratios of CSF concentrations at lumbar versus CM levels were the highest at 4 h, and gradually reduced towards 72 h after IT-L dosing. In addition, the concentration gradients were the highest after IT-L dosing of 1 mg I2S (11.4-, 5.3-, 3.3-, and 1.4-fold at 4, 8, 24, and 48 h, respectively). The respective concentration gradients were 8.4-, 5.7-, 9.8-, 2.9-, and 1.1-fold at 4, 8, 24, 48, and 72 h after IT-L dosing of 10 mg I2S. The lowest gradients were observed after IT-L dosing of 30 mg idursulfase-IT (3.7-, 2.9-, 2.1–1-, and 0.3-fold at 4, 8, 24, 48, and 72 h, respectively). These observations suggested that concentration gradients reduced with escalated IT-L doses.

### Bioavailability of I2S in serum

The values of serum *C*_max_, and *AUC*_inf_ in the 4 groups were normalized for dose (Table 5). The bioavailability was calculated as the percentage of the normalized serum *AUC*_inf_ post IT-L dosing over the normalized serum *AUC*_inf_ post intravenous dosing. Serum bioavailabilities were 40, 52.3, and 82.5%, after IT-L dosing of 1, 10, and 30 mg of idursulfase-IT, respectively, demonstrating an increase in serum bioavailability with increasing dose.

**Table 5:** Bioavailability and exposure fraction (*AUC*_inf_) of I2S in the serum and CSF after IT-L dosing.

	**IV dose**	**IT-L dose**		
	**0.5 mg/kg**	**1 mg**	**10 mg**	**30 mg**
**Serum**	**(n = 7)**	**(n = 4)**	**(n = 6)**	**(n = 7)**
*AUC*_inf_ (all monkeys), h*ng/ml	10,074	2777	37,618	178,042
Mean body weights^a^, kg	2.7	2.9	2.8	2.8
Dose, mg/kg	0.5	0.34	3.57	10.71
*AUC*_inf_ (normalized for dose), h*ng*kg/ml*mg	20,148	8053	10,533	16,617
F (IT-L/IV), %	-	40.0	52.3	82.5
**CSF** ^**b**^	**(n = 3)**	**(n = 4)**	**(n = 4)**	**(n = 4)**
*AUC*_inf_ (CM only), h*ng/ml	390	369,511	4,570,535	16,484,049
Mean body weights^a^, kg	2.7	2.6	2.8	2.7
Dose, mg/kg	0.5	0.38	3.57	11.11
*AUC*_inf_ (normalized for dose), h*ng*kg/ml*mg	780	960,729	1,279,750	1,483,564
EF (IV/IT-L), %	-	0.08	0.06	0.05

*AUC*_inf_, area under the concentration-time curve extrapolated to infinity; CM, cisterna magna; CSF, cerebrospinal fluid; EF, exposure fraction; F, bioavailability; I2S, iduronate-2-sulfatase; IT-L, intrathecal lumbar; IV, intravenous.

^a^Mean body weights were calculated from the animals whose CSF samples were collected at the CM level.

^b^Sampled at CM level.

### Exposure fraction of I2S in CSF

CSF exposure fractions after 0.5 mg/kg intravenous idursulfase dosing were estimated by percentages of the normalized CSF *AUC*_inf_ post-intravenous dosing over the normalized CSF *AUC*_inf_ post-IT-L dosing of idursulfase-IT. The CSF exposure fraction after 0.5 mg/kg intravenous idursulfase dosing was 0.06% compared to 0.08, 0.06, and 0.05% observed following 1-, 10-, and 30- mg IT-L administration (Table 5).

## DISCUSSION

Idursulfase enzyme replacement therapy administered intravenously has been shown to improve walking capacity in patients with Hunter syndrome [4]. Idursulfase has not, however, been specifically evaluated regarding its independent quantifiable impact on CNS pathology, due to the limited permeability of the blood–brain barrier. The IT delivery of idursulfase-IT was used specifically to directly access CNS tissue and thereby overcome the macromolecular distribution limits imposed by the blood–brain barrier.

Studies of other enzymes administered directly to the CNS demonstrate rapid clearance from the CSF [9,10]. Insulin-like growth factor, for example, was completely cleared from the CSF 1 h after intracerebroventricular injection, with consequently little penetration to CNS tissues such as the parenchyma [11]. In contrast, our findings in disease animal models has demonstrated cell uptake of idursulfase-IT by neurons and glial cells, mediated by mannose-6-phosphate receptors and possibly other uptake mechanisms [8].

This study details the pharmacokinetics of I2S in serum and CSF after intravenous or IT-L administration in cynomolgus monkeys. As anticipated, intravenous administration increases the serum concentration of I2S rapidly, although I2S concentrations in CSF are low due to the lack of penetration through the blood–brain barrier. Administration of idursulfase-IT via IT-L also led to increases in serum I2S concentrations with the *C*_max_ occurring 3–4 h after administration. The observed delayed *C*_max_ in the central compartment suggested a slow transition of I2S from CSF to serum. The *C*_max_ for the IT-L dose of 30 mg was greater than the *C*_max_ observed after 0.5 mg/kg intravenous administration (19,971 ng/ml versus 10,029 ng/ml, respectively). The serum I2S bioavailability for the 30 mg IT-L dose was 82.5% of the intravenous dose, suggesting that there is significant flow of I2S from the CSF into the serum.

Administration of idursulfase-IT by the IT-L route, directly bypassing the blood–brain barrier, leads to a rapid increase in CSF I2S concentrations that then declined (compared to C_max_) by approximately 70-, 50-, and 30-fold after 8 h, for the 1-mg, 10-mg, and 30-mg dose groups, respectively, thereafter declining further by first order kinetics. Twenty-four hours after the initial dosing, CSF I2S levels were still significantly higher for all IT-L doses than after 0.5 mg/kg intravenous administration. The *C*_max_ of I2S in the CSF increased in proportion with the idursulfase-IT dose level. There was a slightly greater than dose-proportional response for *AUC*_inf_. The dynamics of I2S transfer in the CSF, evaluated by a pharmacokinetic comparison between CM and lumbar level CSF samples, suggests that there is little, if any, delayed transfer of I2S from lumbar injection region to the CM. Collected from CM level, all animals showed the highest CSF I2S concentration 5 min after IT-L administration (except for 1 animal (014A) in Group 3, *T*_max_ = 15 min).

I2S levels were consistently higher in lumbar samples than in CM samples. It is not immediately clear why, but experience suggests there may be residual protein originating from the dosing port. It remains to be established whether the exposure difference between CM and lumbar samples is due to CSF transport mechanisms, or is due I2S contributed by residual dosing solution.

The CSF/plasma ratio for I2S after IV administration was found to be approximately 0.0387. The value is higher than the literature figures for albumin (0.004–0.006) suggesting a penetration of I2S into CSF slightly greater than that of albumin [12]. The mechanism of I2S translocation from serum into CSF is not yet clear, although receptor-mediated uptake via mannose-6-phosphate receptor (M6PR), or functional leakage of the BBB, or both, could potentially contribute to the I2S translocation from serum to CSF [6]. However, the level of I2S in the CSF after IV administration remained low and is considered not therapeutically relevant.

In general, the pharmacokinetic profiles and the concentration of I2S in the CSF were similar whether the samples were collected at the CM or lumbar levels.

The decrease in CSF concentrations of I2S after IT-L administration was observed to be more rapid when sampling was performed at the CM level compared with sampling performed at the lumbar level. The *t*_1/2_ of I2S in the CSF ranged from 7.8–10 h and from 5.9–6.7 h in samples collected at CM level and lumbar level, respectively. It is not immediately clear why there is a difference in elimination rate of I2S in the CSF between these different regions. The mechanism by which macromolecules translocate from the CNS to the systemic compartment has not yet been clearly elucidated, although it has been reported that the macromolecules may be removed from the CSF through the arachnoid villi [13]. The mean total clearance of I2S from the CSF was 2.3 ml/h, which is consistent with the measured peak CSF flow rate and the rate of CSF replacement reported previously by various authors [14,15,16].

This study was designed to evaluate the pharmacokinetics of I2S in the serum and CSF after IV or IT administration in non-human primates, the purpose of which was to advance our understanding of the clinical feasibility of IT delivery of I2S (idursulfase-IT) in humans. Species differences between cynomolgus and humans, resulting in differences in the systemic disposition of I2S in the CNS cannot be ruled out.

The data presented here demonstrate that IT-L administration effectively delivered I2S to the CSF of cynomolgus monkeys. Although intravenous administration of idursulfase can deliver a high systemic exposure, it does not represent a feasible way to target the CNS. This study suggests that idursulfase-IT enzyme replacement therapy by IT-L administration could deliver I2S to the CNS and in high concentrations. In addition, the long residence time of I2S in the CSF after IT administration should provide the time needed for the I2S to penetrate the brain tissue and degrade the accumulated glycosaminoglycans, which may have an impact on the progression of CNS disease manifestations in patients with Hunter syndrome. The precise disposition of I2S, following IT-L administration, at the tissue and cellular levels within the CNS is still unclear and more studies, using imaging technology or immunohistochemistry, are needed to help understand this.

## References

[1] Meikle PJ, Hopwood JJ, Clague AE, Carey WF (1999) Prevalence of lysosomal storage disorders. JAMA 281: 249–254. 9918480 10.1001/jama.281.3.249

[2] Bach G, Eisenberg F Jr, Cantz M, Neufeld EF (1973) The defect in the Hunter syndrome: deficiency of sulfoiduronate sulfatase. Proc Natl Acad Sci U S A 70: 2134–2138. 4269173 10.1073/pnas.70.7.2134 PMC433682

[3] Neufeld EF, Muenzer J (2001) The mucopolysaccharidoses In: Scriver CR, Beaudet AL, Sly WS, Valle D, editors. The Metabolic and Molecular Bases of Inherited Disease, Volume III 8 ed. New York: McGraw-Hill pp. 3421–3452.

[4] Muenzer J, Wraith JE, Beck M, Giugliani R, Harmatz P, Eng CM, et al (2006) A phase II/III clinical study of enzyme replacement therapy with idursulfase in mucopolysaccharidosis II (Hunter syndrome). Genet Med 8: 465–473. 10.1097/01.gim.0000232477.37660.fb 16912578

[5] Wraith JE, Scarpa M, Beck M, Bodamer OA, De Meirleir L, Guffon N, et al (2008) Mucopolysaccharidosis type II (Hunter syndrome): a clinical review and recommendations for treatment in the era of enzyme replacement therapy. Eur J Pediatr 167: 267–277. 10.1007/s00431-007-0635-4 18038146 PMC2234442

[6] Felice BR, Wright TL, Boyd RB, Butt MT, Pfeifer RW, Pan J, et al (2011) Safety evaluation of chronic intrathecal administration of idursulfase-IT in cynomolgus monkeys. Toxicol Pathol 39: 879–892. 10.1177/0192623311409595 21628718

[7] Hemsley KM, Hopwood JJ (2009) Delivery of recombinant proteins via the cerebrospinal fluid as a therapy option for neurodegenerative lysosomal storage diseases. Int J Clin Pharmacol Ther 47 (Suppl 1): 118S–123S. 10.5414/cpp47118 20040322

[8] Calias P, Papisov M, Pan J, Savioli N, Belov V, Huang Y, et al (2012) CNS penetration of intrathecal-lumbar idursulfase in the monkey, dog and mouse: implications for neurological outcomes of lysosomal storage disorder. PLoS One 7: e30341 10.1371/journal.pone.0030341 22279584 PMC3261205

[9] Ghersi-Egea JF, Gorevic PD, Ghiso J, Frangione B, Patlak CS, Fenstermacher JD (1996) Fate of cerebrospinal fluid-borne amyloid beta-peptide: rapid clearance into blood and appreciable accumulation by cerebral arteries. J Neurochem 67: 880–883. 8764620 10.1046/j.1471-4159.1996.67020880.x

[10] Greene HL, Hug G, Schubert WK (1969) Metachromatic leukodystrophy. Treatment with arylsulfatase-A. Arch Neurol 20: 147–153. 5818425 10.1001/archneur.1969.00480080047005

[11] Nagaraja TN, Patel P, Gorski M, Gorevic PD, Patlak CS, Fenstermacher JD (2005) In normal rat, intraventricularly administered insulin-like growth factor-1 is rapidly cleared from CSF with limited distribution into brain. Cerebrospinal Fluid Res 2: 5 10.1186/1743-8454-2-5 16045806 PMC1190198

[12] Andersson M, Alvarez-Cermeno J, Bernardi G, Cogato I, Fredman P, Frederiksen J, et al (1994) Cerebrospinal fluid in the diagnosis of multiple sclerosis: a consensus report. J Neurol Neurosurg Psychiatry 57: 897–902. 8057110 10.1136/jnnp.57.8.897 PMC1073070

[13] Bernards C (1999) The spinal meninges and their role in spinal drug movement In: Yaksh TL, editor. Spinal Drug Delivery. Amsterdam: Elsevier pp. 634.

[14] Kapoor KG, Katz SE, Grzybowski DM, Lubow M (2008) Cerebrospinal fluid outflow: an evolving perspective. Brain Res Bull 77: 327–334. 10.1016/j.brainresbull.2008.08.009 18793703

[15] Loth F, Yardimci MA, Alperin N (2001) Hydrodynamic modeling of cerebrospinal fluid motion within the spinal cavity. J Biomech Eng 123: 71–79. 11277305 10.1115/1.1336144

[16] Yoshida K, Takahashi H, Saijo M, Ueguchi T, Tanaka H, et al (2009) Phase-contrast MR studies of CSF flow rate in the cerebral aqueduct and cervical subarachnoid space with correlation-based segmentation. Magn Reson Med Sci 8: 91–100. 19783872 10.2463/mrms.8.91

